# The Relationship Between Systemic Immune Inflammatory Index and Prognosis of Patients With Non-Small Cell Lung Cancer: A Meta-Analysis and Systematic Review

**DOI:** 10.3389/fsurg.2022.898304

**Published:** 2022-06-30

**Authors:** Wei Huang, Jiayu Luo, Jianbo Wen, Mingjun Jiang

**Affiliations:** ^1^ Department of Cardiothoracic Surgery, The Affiliated People’s Hospital of Ningbo University, Ningbo, China; ^2^ Department of Oncology, No.906 Hospital of People’s Liberation Army, Ningbo, China

**Keywords:** systemic immune inflammatory index, non-small cell lung cancer, prognosis, meta-analysis, systematic review

## Abstract

**Background:**

The relationship between systemic immune inflammation index (SII) and the prognosis of cancer has always been a subject of intense interest. However, the prognostic value of SII in non-small cell lung cancer (NSCLC) patients remains a controversial topic.

**Objective:**

To evaluate the effect of SII index on prognosis of NSCLC.

**Methods:**

We conducted a comprehensive search of PubMed, EMBASE, and the Cochrane Library databases to determine correlation between SII index, clinicopathological features, overall survival (OS), and progression-free survival (PFS). Odds ratio (ORs) and 95% confidence interval (CIs) were used to assess the connection between SII and clinicopathological parameters, and HRs and 95% CIs were used to assess the connection between SII and survival.

**Results:**

Seventeen studies with 8,877 cases were included in the analysis. Compared with NSCLC patients with low SII level, patients with NSCLC with high SII level had a poor OS (HR = 1.75, 95% CI, 1.50–2.00; *P* < 0.001) and had a poor PFS (HR = 1.61, 95% CI, 1.25–1.96; *P* < 0.001). In addition, patients with higher pathological stage (II–III) had higher SII levels (OR = 2.32, 95% CI, 2.06–2.62; *P* < 0.001).

**Conclusions:**

The SII index is a promising prognostic biomarker for NSCLC and may help clinicians choose appropriate NSCLC treatments.

## Introduction

Lung cancer has a high incidence and mortality, and non-small cell lung cancer (NSCLC) accounts for about 80% of the total incidence of lung cancer (1). The incidence of NSCLC has risen steadily over the past few decades, but the mortality rate of NSCLC appears to be decreasing, possibly due to the tremendous advances in NSCLC treatment (2, 3). The treatment of NSCLC includes surgery, radiotherapy, chemotherapy, targeted therapy, and immunotherapy (4). Nonetheless, the efficacy of these therapies in NSCLC patients remains dissatisfactory due to the lack of resultful indicators that can be utilized to predict the disease course and the widespread chemoresistance of NSCLC (5). Hence, it is necessary for researchers to identify exact biomarkers and potential therapeutic targets for NSCLC to improve survival.

In recent years, some indicators reflecting the inflammatory state of the body have been confirmed to be related to the prognosis of various malignant tumors (6). Systemic immune inflammation index (SII) is one of the new inflammatory indexes based on peripheral blood platelet count, neutrophils, and lymphocytes. SII = platelet count × neutrophils/lymphocytes. Studies have confirmed that SII can impersonally reflect the balance between inflammatory response and immune response in tumor patients (7). SII has achieved good results in predicting the prognosis of colorectal cancer, cervical cancer, pancreatic cancer and other malignant tumors (8–10). Furthermore, multiple meta-analyses have shown that SII predicts poor prognosis in a variety of malignancies (11, 12). Nevertheless, studies on SII levels in NSCLC are limited, and the prognostic value of SII levels in NSCLC is still a controversial issue. To solve this problem, we conducted a meta-analysis to synthetically assess the value of SII as a prognostic marker and determine the correlation between SII levels and pathological characteristics of NSCLC patients.

## Materials and Methods

This meta-analysis was based on preferred reporting items for systematic reviews and meta-analysis (PRISMA) (13). This study was based on previously published research data, ethical approval is not necessary.

### Literature Search

We performed a comprehensive literature search of published studies using databases such as PubMed, EMBASE, and Cochrane. Studies published before January 2022 were collected. The following keywords were used in the search box: (“Systemic immune inflammation index” OR “SII” OR “neutrophil × platelets/lymphocyte” OR “platelet count × NLR”) AND (“non small cell lung cancer” OR “NSCLC”). In order to find relevant literature, we also checked the references of relevant articles.

### Inclusion and Exclusion Criteria

Studies that met the following conditions were considered eligible: (1) The relationship between SII index and survival of NSCLC patients was provided. (2) The critical value of preprocessing SII was provided. (3) The study offered enough data to extract the hazard ratio (HR) and 95% confidence interval (CI) for OS. Articles were excluded if they were reviews or meta-analyses, did not involve non-small cell lung cancer, or only involved animal experiments. If duplicate articles exist, only complete or up-to-date articles were included in this analysis.

### Data Extraction and Quality Assessment

All relevant data will be extracted by two data collectors, and if the two collectors were unsure of the data, one researcher wound decided how to extract the data. First author, country, number of cases, patient age, SII index, clinicopathological parameters, HR, and 95% CI were extracted from each study. Quality assessment of each study was performed independently by two data collectors using the Newcastle-Ottawa Scale (NOS), and the quality score was averaged between the two data collectors. The highest NOS score was 9, and studies with a score greater than 6 were considered high quality (14).

### Statistical Methods

HRs and their 95% CIs were used to assess the correlation between SII and survival, and Odds ratio (ORs) and their 95% confidence interval (CIs) were used to determine the correlation between SII and clinicopathological parameters. Heterogeneity between studies was assessed using the chi-square test and I^2^. Statistics *P* < 0.1 or I^2^ > 50% indicated significant heterogeneity between studies and a random effects model (REM) was selected for analysis; otherwise, a fixed effects model (FEM) was selected for analysis. Egger’s test was selected for assess potential publication bias. Review Manager 5.3 (Revman the Cochrane Collaboration) and STATA 16.0 (Stata Corporation) software were selected for Meta-analysis. *P* < 0.05 means the difference is obviously significant. *P* values and 95% CI were two-sided tests.

## Results

### Search Results and Study Characteristics

**Figure 1:**
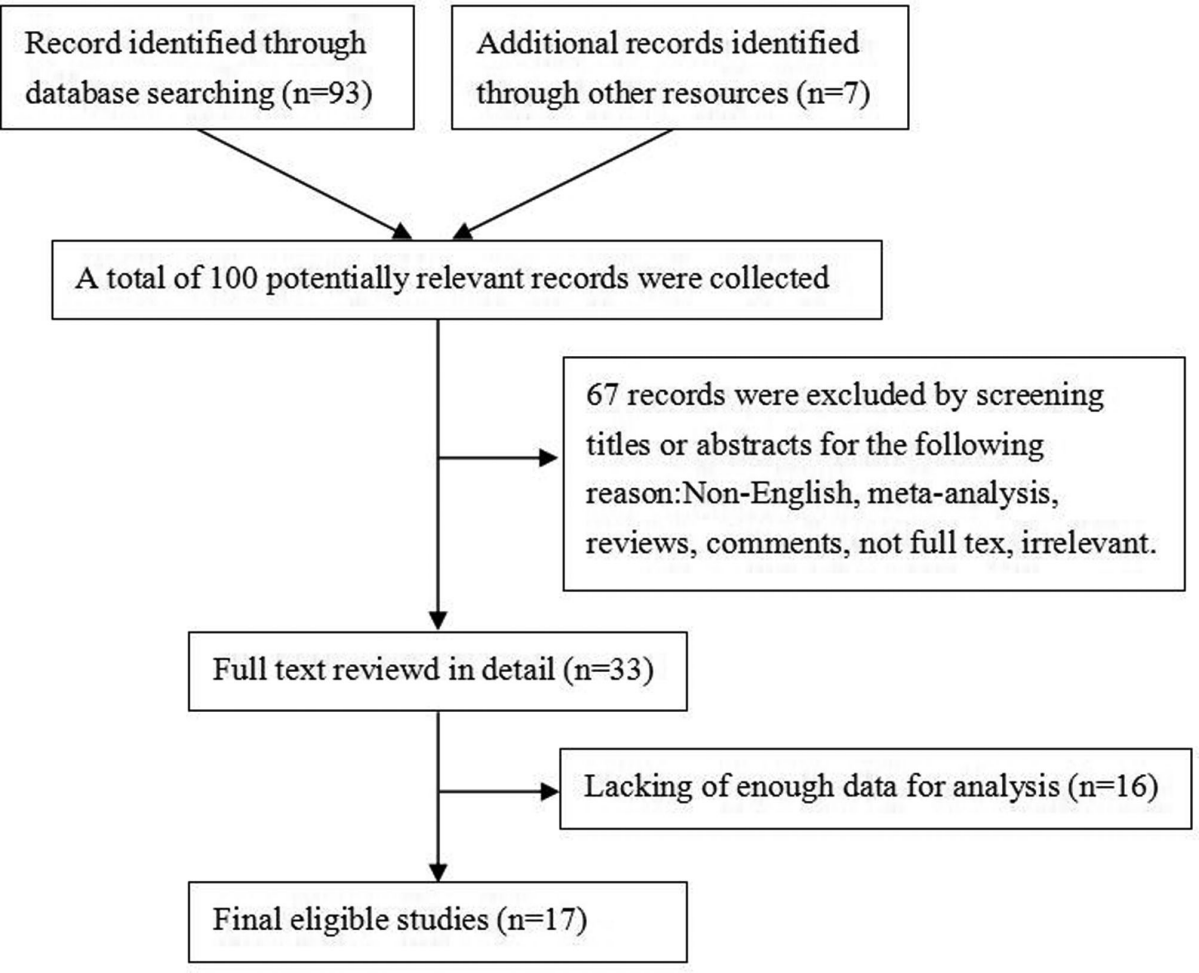
Literature screening process.

In this study, we collected 100 total potentially relevant articles according to the search methods previously determined. After reviewing the titles and abstracts of these articles, we excluded 67 duplicate or irrelevant studies. After a detailed review of 33 articles, we determined that 17 trials accord with inclusion criteria and were therefore included in the meta-analysis (as shown in Figure 1).

The information of the 17 studies (15–33) are shown in Table 1. Of the 17 studies, the sample size was a minimum of 42 and a maximum of 3,984. 8,877 total patients participated in the study. A total of 17 studies eligible for analysis were retrospective. Four of the studies were from the United States and Japan, and the rest were from China. Nine studies were conducted with patients with advanced stage, and the remaining studies were conducted with patients with early and advanced stage. HRs and 95% CIs were extracted directly from all original articles. The quality of the studies assessed by NOS was all ≥6. Therefore, the study was of high quality.

**Table 1:** Characteristics of included studies.

First author/year	Country	Total	male ratio	Age Median(range)	Survival type	Cut off value(×10^9^/L)	Cut off selection	Group size	Tumor stage	NOS
Berardi R 2019	USA	311	216(69%)	68(25–86)	PFS,OS	1,270	median	High 179 /low 132	III–IV	7
Chen X 2022	China	94	55 (58.5%)	48(18–76)	PFS,OS	842	median	high 47 /low 47	IIIB–IV	6
Deng C 2019	China	203	89 (43.8%)	59(28–79)	PFS,OS	1,066.935	ROC curve analyses	high 63 /low 140	–	7
Fu F 2021	China	3,984	2,139 (53.7%)	60 (53–66)	PFS,OS	479	R package survminer	high 1,643 /low 2,341	I–III	7
Gao Y 2018	China	410	267(65.12%)	–	OS	395.4	ROC curve analyses	high 270 /low 140	T1–T4	7
Guo D 2018	China	140	95 (67.9%)	62(33–83)	PFS,OS	521	ROC curve analyses	high 72 /low 68	IIIB – IV	7
Guo W	China	569	425 (74.7%)	60(27–80)	OS	419.6	ROC curve analyses	high 307 /low 262	I–III	7
Hong X 2015	China	919	635 (69.1%)	56(16–84)	OS	1,600	ROC curve analyses	high 127 /low 792	I–IV	7
Ju Q 2021	China	102	41 (40.2%)	59.50(30–80)	PFS,OS	841.03	ROC curve analyses	NA	III–IV	6
Keit E 2021	USA	125	64 (51.2%)	67(45–86)	PFS,OS	1,266	ROC curve analyses	high 55 /low 70	III	7
Li A 2020	China	252	145(57.5%)	58 (24–84)	OS	630.85	ROC curve analyses	high 154 /low 98	Brain metastasis	7
Li X 2020	China	345	255(73.9%)	64 (25–93)	OS	555.59	ROC curve analyses	high 196 /low 149	IIIB – IV	7
Takeda T 2021	Japan	42	22(52.4%)	67(29–85)	PFS	1,000	ROC curve analyses	high 15 /low 27	I–III	7
Tong YS 2017	China	332	206 (62%)	61(34–70)	OS	660	ROC curve analyses	high 149 /low 183	IIIA – IIIB	7
Watanabe K 2021	Japan	387	233(60.2%)	71(19–86)	RFS	715	ROC curve analyses	high 97 /low 290	IA–IIA	7
Yan X 2020	China	538	343 (63.8%)	60 (24–82)	DFS,OS	402.37	ROC curve analyses	high 339 /low 199	I–IIIA	7
Zhang Y 2021	China	124	56(45.2%)	60 (38–73)	PFS,OS	480	ROC curve analyses	high 66 /low 58	I–III	7

### Relationship Between SII Levels and OS

**Figure 2:**
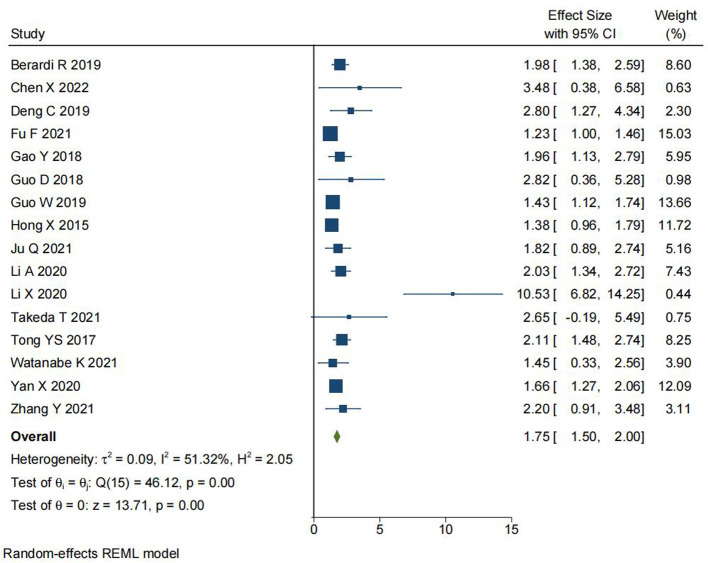
Forest plot depicting the relationship between SII levels and OS in NSCLC.

We analyzed the relationship between SII levels and overall survival (OS) in NSCLC. 8,752 total cases were included in 16 studies. The meta-analysis (as shown in Figure 2) demonstrated that NSCLC patients with high-level SII had a poor OS contrast to NSCLC patients with low-level SII (HR = 1.75, 95% CI, 1.50–2.00; *P* < 0.001). There was heterogeneity in the results (I^2^ = 51.32%, *P* < 0.001), hence, a REM was used for analysis.

### Correlation of SII Levels With PFS

**Figure 3:**
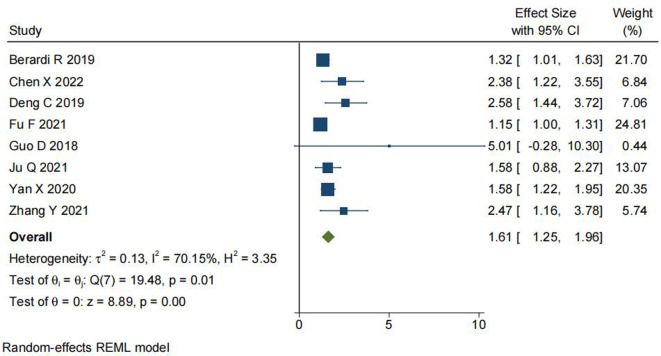
Forest plot depicting the relationship between SII levels and PFS in NSCLC.

We analyzed the relationship between SII levels and progression-free survival (PFS) in NSCLC. Eight studies included a total of 5,496 patients. The meta-analysis (as shown in Figure 3) demonstrated that NSCLC patients with high-level SII had a poor PFS contrast to NSCLC patients with low-level SII (HR = 1.61, 95% CI, 1.25–1.96; *P* < 0.001). There was heterogeneity in the results (I^2^ = 70.15%, *P* = 0.01), hence, a REM was used for analysis.

### Relationship Between SII Levels and Clinicopathological Features

**Figure 4:**
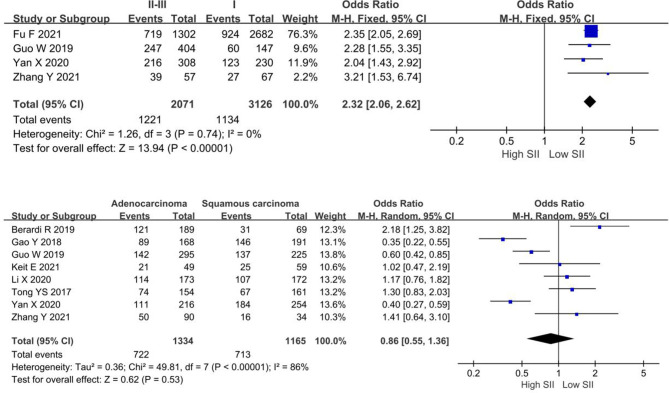
Forest plot depicting the relationship between SII levels and clinicopathological characteristics of NSCLC patients.

In this study, we analyzed the relationship between high levels of SII and pathological stage and pathological type. The meta-analysis demonstrated that patients with higher pathological stage (II–III) had higher SII levels (OR = 2.32, 95% CI, 2.06–2.62; *P* < 0.0001). However, no obvious correlation between SII levels and pathological types (OR = 0.86, 95% CI, 0.55–1.36, *P* = 0.53), as shown in Figure 4.

### Sensitivity Analysis

Sensitivity analysis, which involves deleting one study at a time to assess the stability of the results. After removing the literature, none of the individual studies obviously affected the whole population, Indicating that the results of the current meta-analysis were credible.

### Publication Bias

**Figure 5:**
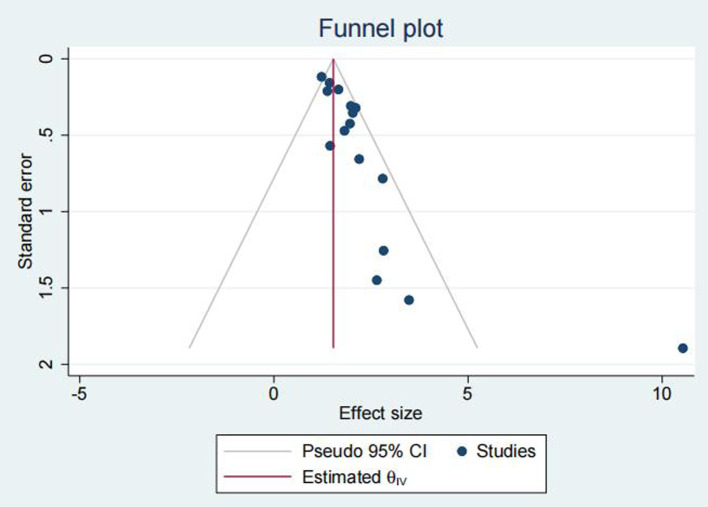
Funnel plot, publication bias assessment.

Egger’s test indicated that the included studies exhibited publication bias affecting the hazard ratio for OS, with a *P* value of 0.0001, as shown in Figure 5.

## Discussion

A large number of studies have reported the relationship between inflammation and tumor and found that inflammation is one of the factors promoting the occurrence and development of tumor (34). For example, neutrophils, lymphocytes, and platelets play important roles in tumor progression. These indicators can promote tumor cell proliferation, invasion, and distant metastasis (35). For the past few years, some systemic inflammatory cell-based biomarkers, such as platelet-to-lymphocyte ratio (PLR) and neutrophil-to-lymphocyte ratio (NLR), have been shown to correlate with many The prognosis of this type of cancer is relevant (36). Nevertheless, these indicators are based on two inflammatory indices, and SII is a novel biomarker based on three indices (platelet, neutrophil, lymphocyte count) that comprehensively reflects the host immune and inflammatory status. SII is a relatively objective index and has good prognostic reliability (8–10).

As far as we know, a meta-analysis of the effect of SII index on survival in patients with NSCLC has not been reported. In this context, a comprehensive literature search was conducted and incorporated into 17 total studies with 8,877 cases. From the results of the meta-analysis, we found a obvious correlation between the SII index and the prognosis of NSCLC patients. The OS and PFS of patients with high SII levels were shorter than those with low SII levels, suggesting that SII index may be a promising prognostic factor for NSCLC patients. Berardi et al. (15), Deng et al. (17), Hong et al. (22), Li et al. (26), Tong et al. (29), Yucel et al. (32) and other results demonstrated that the median OS of patients with high SII level was obviously lower than that of patients with low SII level. In addition, this meta-analysis also observed that the SII index of NSCLC patients with higher pathological stages (II–III) was significantly higher than that of stage I patients, suggesting that SII index may be a risk factor for disease development in patients with NSCLC.

We tried to do a comprehensive analysis, but there are still limitations to this study. First, treatment strategies not analyzed in this research may have influenced the results. Second, the included studies were limited to articles published in English and mostly from China, which may lead to publication bias. Third, the cutoff values used to determine high levels of SII also inconsistent. Finally, the sample size of the studies included in the analysis may have contributed to heterogeneity. However, this meta-analysis has proved an correlation between high levels of SII and clinicopathological factors in NSCLC. The results of this study may improve the prognosis of NSCLC. However, despite the robustness of our findings, caution is required in interpreting the validity of SII in NSCLC prognosis.

In conclusion, this meta-analysis identified pretreatment or preoperative SII index as a prognostic factor for OS and PFS in NSCLC patients. SII index may be an effective survival indicator of NSCLC. Larger multicenter studies are needed in the future to further verify the clinical application value of the SII index.

## Data Availability

The original contributions presented in the study are included in the article/Supplementary Material, further inquiries can be directed to the corresponding author/s.
